# Vertebrate SLRP family evolution and the subfunctionalization of osteoglycin gene duplicates in teleost fish

**DOI:** 10.1186/s12862-018-1310-2

**Published:** 2018-12-13

**Authors:** R. A. Costa, R. S. T. Martins, E. Capilla, L. Anjos, D. M. Power

**Affiliations:** 1 Comparative Endocrinology and Integrative Biology Group, Centre of Marine Sciences, University of Algarve, Campus of Gambelas, 8005–139 Faro, Portugal; 2 Department of Cell Biology, Physiology and Immunology, Faculty of Biology, University of Barcelona, 08028 Barcelona, Spain

**Keywords:** Small leucine-rich proteoglycan (SLRP) family, Osteoglycin, Osteoblasts, Myocytes, Adipocytes, Teleosts

## Abstract

**Background:**

Osteoglycin (OGN, a.k.a. mimecan) belongs to cluster III of the small leucine-rich proteoglycans (SLRP) of the extracellular matrix (ECM). In vertebrates OGN is a characteristic ECM protein of bone. In the present study we explore the evolution of SLRP III and OGN in teleosts that have a skeleton adapted to an aquatic environment.

**Results:**

The SLRP gene family has been conserved since the separation of chondrichthyes and osteichthyes. Few gene duplicates of the SLRP III family exist even in the teleosts that experienced a specific whole genome duplication. One exception is *ogn* for which duplicate copies were identified in fish genomes. The *ogn* promoter sequence and in vitro mesenchymal stem cell (MSC) cultures suggest the duplicate *ogn* genes acquired divergent functions. In gilthead sea bream (*Sparus aurata*) *ogn1* was up-regulated during osteoblast and myocyte differentiation in vitro, while *ogn2* was severely down-regulated during bone-derived MSCs differentiation into adipocytes in vitro.

**Conclusions:**

Overall, the phylogenetic analysis indicates that the SLRP III family in vertebrates has been under conservative evolutionary pressure. The retention of the *ogn* gene duplicates in teleosts was linked with the acquisition of different functions. The acquisition by OGN of functions other than that of a bone ECM protein occurred early in the vertebrate lineage.

**Electronic supplementary material:**

The online version of this article (10.1186/s12862-018-1310-2) contains supplementary material, which is available to authorized users.

## Highlights

- Subfunctionalization of duplicate osteoglycin genes (*ogn1* and *ogn2*) occurred during teleost evolution;
- *Ogn1* transcripts are up-regulated in the early stages of osteoblast and myocyte differentiation in vitro;
- *Ogn2* transcripts are down-regulated in bone-derived MSCs under osteoinductive and adipogenic conditions;

## Background

The extracellular matrix (ECM) is important in multicellular organisms and establishes the basic characteristics of each tissue [1]. The essential building blocks of the ECM are ubiquitous across organisms and include collagens, glycoproteins and proteoglycans [2–4]. The increased ECM complexity in terrestrial and aquatic vertebrates relative to early chordates is associated with gene family expansion through duplication of ancestral metazoan genes, and through a small number of vertebrate specific gene innovations [1]. Knowledge about the ECM in fishes is very patchy despite their unique adaptations and their evolutionary success (there are over 34,000 extant species) [5]. Furthermore, the increased gene number due to teleost specific gene duplications not only elevates the number of potential genes involved in the ECM but also the scope for gene innovations [6–8].

The proteoglycans are grouped into 4 major classes based on their cellular and subcellular location, overall gene/protein homology, and the presence of specific protein modules [3]. The small leucine-rich proteoglycan (SLRP) family comprises the largest class of proteoglycans in the ECM. They are extracellular proteins with a small protein core, harbouring tandem leucine-rich repeats (LRRs) that may contain one or more glycosaminoglycan side chains, although there are some exceptions [9, 10]. The SLRP family is clustered into 5 main groups (cluster I-V) when protein and gene homology, chromosome localization and the presence and spacing of the classical N-terminal cysteine-rich repeats are considered [1, 11–13]. The SLRPs have a diversity of functions that depend on tissue context and the specific characteristics of the organism. Functional compensation can occur between SLRPs and an example of this is the up-regulation of decorin when biglycan is lost in humans [14].

The present study is focused on osteoglycin (OGN, a.k.a. mimecan) that belongs to SLRP cluster III together with epiphycan (EPYC) and opticin (OPTC) [11]. Members of cluster III are characterized by a low number of LRRs (relative to other SLRP classes) and an N-terminal consensus sequence for tyrosine sulphation [15]. The function of OGN has mainly been studied in mammals in which it regulates collagen fibrillogenesis, the efficiency of which is increased when it is processed by BMP-1/Tolloid-like metalloproteinases [16]. OGN KO-mice are viable, fertile and grow normally but the skin has a modified tensile strength due to abnormalities in the collagen fibrils, which are on average thicker [17]. OGN has a role in wound healing in the cornea, in atherosclerotic lesions and modulates myocardial integrity and remodelling [17–20]. In addition, OGN enhances the neurite outgrowth promoted by insulin-like growth factor-2 and IGF binding protein-2 [21]. The presence of *OGN* in the mouse and human pituitary gland co-expressed with proopiomelanocortin and its up-regulation by glucocorticoids and adrenocorticotropic hormone reveals a novel function for OGN in the hypothalamic-pituitary-adrenal axis in mammals [22, 23]. An emerging role for OGN secreted by adipose tissue is its action as a satiety factor acting at the level of the hypothalamus [24]. OGN is also implicated in several pathologies and is down-regulated in tissues derived from colorectal adenomas and cancers when compared to normal mucosa [25].

Although relatively well characterized in mammals, far less is known about the function of OGN in other vertebrate groups, including fish. In this respect, the up-regulation of *ogn* transcription and protein levels in the gilthead sea bream (*Sparus aurata*), 4–5 days after scale removal, suggests a role in skin and scale regeneration [26, 27]. In Atlantic salmon (*Salmo salar*) *ogn* transcript levels were significantly down-regulated in the skin of fish exposed to increased cortisol for 18 days, further suggesting a role in skin physiology [28]. In the blunt snout bream (*Megalobrama amblycephala*) *ogn* is involved in the growth of intermuscular bone [29]. Furthermore, in zebrafish (*Danio rerio*) Ogn levels increase significantly during caudal fin regeneration and skeletal development suggesting its involvement in bone and skeletal development [30].

In the present study we aimed to understand the evolution of teleost *ogn* in the context of SLRP family evolution. So far, a role for OGN in bone seems to be common across vertebrates but in mammals, several other functions have been described. To assess if the diversification of OGN function was an innovation of the terrestrial vertebrates we used bioinformatics to analyse the *ogn* promoter. We characterized gene expression in bone and muscle cell differentiation from gilthead sea bream (*Sparus aurata*) mesenchymal stem cells (MSCs). The gilthead seabream was chosen for the study as: i) it is an important Mediterranean aquaculture species, which frequently presents skeletal malformations, ii) it is a representative of the Perciformes, a group that underwent a large radiation and contains other aquaculture species and iii) its size facilitates establishment of primary cell cultures. Overall, the results indicate gene conservation during evolution and retention of the duplicate *ogn* genes that arose during the teleost specific whole genome duplication. Evidence for subfunctionalization of the duplicate teleost *ogn*s was uncovered and a role as a candidate factor in early differentiation of multiple cell types was demonstrated.

## Methods

### Identification and characterization of the osteoglycin (*ogn*) gene(s) in gilthead sea bream

To identify homologue(s) of *ogn* in the gilthead sea bream (*S. aurata*) available transcriptome data from vertebra, gill arch [8] and white muscle [31] was searched using the BLAST algorithm and human *ogn* cDNA (NP_054776) as the bait. *Ogn* homologues were extracted from the genome of terrestrial vertebrates (placental mammals, ungulates, birds and amphibians) and aquatic vertebrates (turtles, marine mammals and fish) using sequence similarity searches (BLASTX and TBLASTN) [32] in NCBI (http://www.ncbi.nlm.nih.gov/), Ensembl [33] and the European sea bass genome (http://seabass.mpipz.de/ version dicLab v1.0c) [34] databases. OGN sequences were aligned using ClustalX (v2.0.11) [35], the alignments edited and the percentage of protein sequence similarity between OGN homologues determined using GeneDoc version 2.7.0 [36]. The accession numbers of all the sequences used are indicated in Additional file 1.

Preliminary data about *ogn* tissue distribution in teleosts was obtained by carrying out sequence similarity searches (BLASTX and TBLASTN) against the Expressed Sequence Tags (ESTs) database [37].

### Phylogenetic analysis and gene environment

In this study we first identified the gene repertoire of vertebrate SLRP members in order to, i) ensure the correct clustering of gilthead sea bream Ogn sequences, and ii) to further characterize when and how *ogn* genes duplicated in fish. To achieve the first objective, we used the sequences of the 18 known SLRPs in the human genome to search for putative orthologues in sharks (*Callorhynchus milii* and *Rhincodon typus*), spotted gar (*Lepisosteus oculatus*), African coelacanth (*Latimeria chalumnae*) and representatives of teleost fish (Cypriniformes: *Danio rerio* and Perciformes: *Dicentrarchus labrax*). To improve resolution of the relationship between the ECM like genes we also included the SLRP sequences of other tetrapods (*Xenopus tropicalis, Anolis carolinensis, Gallus gallus, Mus mus and Sarcophilus harrisii*) and teleost fish from different orders (Pleuronectiformes: *Paralichthys olivaceus*; Beloniformes: *Oryzias latipes*; Cichliformes: *Oreochromis niloticus*; Characiformes: *Astyanax mexicanus, Pygocentrus nattereri*; Siluriformes: *Ictalurus punctatus*; Salmoniformes: *Salmo salar* and Cypriniformes: *Cyprinus carpio, Sinocyclocheilus graham*) (see Additional file 1 for accession numbers). These sequences were then used for phylogenetic analysis using the leucine-rich repeat and immunoglobulin-like domain-containing nogo receptor interacting protein 3 (Lingo 3) from *Rhincodon typus* as an out-group (accession number: XP_020369864). The full-length, deduced protein sequence of SLRP members were used in multiple sequence alignments (using ClustalW v.2.0) [33] and were analysed with ProtTEST (v2.4) [38] to select the model of protein evolution that best fit the dataset. For the SLRP family, the ATGC interface was used (PhyML 3.0) [39] and the ML phylogenetic method was applied with 100 bootstrap replicates using a JTT substitution model and a discrete gamma distribution of rates among sites with 4 categories.

Clustering of the SLRPs identified the sequences assigned to OGN and these were then used for in depth phylogenetic analysis using all other SLRP family members as the out-group. Phylogenetic analysis was performed using Bayesian inference and the tree was built in MrBayes 3.2 [40] using a JTT substitution model (model = Jones) [41] and 1.000.000 sampling generations to obtain the probability values to support the tree branching. The accession numbers of all the sequences used to generate the phylogenetic trees are indicated in Additional file 1.

A branch-specific test to detect signatures of natural selection in vertebrate OGNs, was used to assess the presence of significantly divergent branches in the ML gene tree (Branch Site REL) [42]. The full-length, vertebrate *OGN* nucleotide coding sequences aligned in ClustalX v2.0.11 [35] (see Additional file 2 for accession numbers) were transferred into Translator X [43] to obtain a codon-based alignment. The user tree option for analysis in Data Monkey (http://www.datamonkey.org/) of branch- and site-specific codon evolution was the vertebrate *OGN* ML tree.

To identify the gene environment of fish *ogn* duplicates and compare it with the gene environment of *OGN* from other vertebrates, short-range synteny analysis was performed. The genes that flank *ogn1* (LG1A: 25953681–25,958,751) and *ogn2* (LG22–25: 8776719–8,778,287) were retrieved from the European sea bass (*Dicentrarchus labrax*), Japanese puffer fish (*Takifugu rubripes*) (*ogn1* scaffold_192: 347893–352,120 and *ogn2* scaffold_75: 1027406–1,028,311), zebrafish (*Danio rerio*) (*ogn1* Chr.22: 10564753–10,570,030 and *ogn2* Chr.23: 19968107–19,971,521), spotted gar (*Lepisosteus oculatus*) (*ogn1* LG5: 46192316–46,202,415), coelacanth (*Latimeria chalumnae*) (*ogn1* JH126569.1: 5130733–5,135,069) and human *OGN* (Chr. 9: 92389641–92,393,152).

### Promoter analysis

To assess if the divergent expression of *ogn1* and *ogn2* was a consequence of divergent regulation at the level of the promoters, the sea bass (http://seabass.mpipz.de/ version dicLab v1.0c) [34], which shares evolutionary proximity with the gilthead sea bream and has a fully sequenced genome, was used. A 2 Kb sequence upstream of sea bass *ogn1* (DLAgn_00097140, LG1A: 25958752–25,960,751) and *ogn2* (DLAgn_00129310, LG22–25:8774557–8,776,556) was extracted and the transcription start site and the putative transcription factor binding sites for each gene was identified using MatInspector [44]. Position weight matrices were used to represent the transcription factor binding sites using the default parameters. The matrix family library Version 9.1 of the genomatix suite (http://www.genomatix.de) was used for the analysis.

### Multiple sequence alignments and protein characterization

A multiple sequence alignment (ClustalX v2.0.11) [35] of the deduced amino acid sequences for fish (*Lepisosteus oculatus, Sparus aurata, Oreochromis niloticus, Gadus morhua, Danio rerio, Latimeria chalumnae*) and terrestrial vertebrate (*Xenopus tropicalis, Gallus gallus, Homo sapiens*) OGNs was used to identify conserved motifs and domains using UniProt [45], PROSITE [46], InterPro [47] and Pfam [48] databases (see Additional file 1 for accession numbers).

The consensus sequence for LRR repeat motifs characteristic of class III SLRP family members were identified manually (LXXLXLXXN/CXL, where L is a hydrophobic amino acid, N is Asn and C is Cys, and X is any amino acid). The characteristic N-terminal (CX_2_CXCX_6_C) and C-terminal (CX_33_C) cysteine-rich clusters and disulphide bonds were identified by sequence similarity with annotated OGN sequences using DISULFIND software v.4. [49]. The signal peptide, molecular weight and isoelectric point of predicted proteins were determined using SignalP v.4.1 [50] and ProtParam [51]. Post-translational modification (PTM) sites were also identified [52–57]. PROSITE MyDomains image creator software [58] was used to build representative OGN structures.

### Animal experiments

#### Ethics statement

The maintenance of the fish and subsequent experiments carried out at Ramalhete, the experimental station of the Centre of Marine Sciences (CCMAR, University of Algarve, Faro, Portugal) and at the University of Barcelona (UB, Barcelona, Spain) complied with the Guidelines of the European Union Council (86/609/EU) and were covered by a group 1 license (Direção-Geral de Veterinária, Portugal) or approved by the corresponding Ethics and Animal Care Committee of Barcelona (permit numbers CEEA 243/12 and DAAM 6759). The behaviour and health of all animals was monitored daily and no evidence of infection, modified behaviour or mortality was observed during the experiments.

#### Tissue sampling

Gilthead sea bream juveniles (*N* = 3; 94–140 g) maintained under standard conditions (500 L open circuit sea water tanks, see below for details) at CCMAR, were anesthetized with 2-phenoxyethanol (1:10,000; Sigma-Aldrich, Três Cantos, Spain) and then killed. Nine tissues (fast-twitch/white skeletal muscle, visceral adipose tissue, vertebra, kidney, gill arches, gill filaments, skin, heart and liver) were collected and immediately snap-frozen in liquid nitrogen. The tissue panel was used to assess the distribution of *ogn* using quantitative real-time PCR (qPCR).

#### Tissue culture experiments

Gilthead sea bream juveniles were obtained from a hatchery in Northern Spain and maintained in the animal facility of the Faculty of Biology at the University of Barcelona in 200 L fiberglass tanks at 21 ± 1 °C, pH 7.5–8, 31–38 ‰ salinity and > 80% oxygen saturation under a 12 h light/12 h dark photoperiod and fed ad libitum twice daily with a commercial feed (Excel; Skretting, Burgos, Spain).

#### Bone-derived MSCs gilthead sea bream primary culture

Primary cultures (*N* = 5) derived from vertebra of gilthead sea bream juveniles (8–38 g) were performed using an established protocol [59]. Briefly, the vertebral columns of 6 fish per culture were removed, washed and chopped-up into small fragments with a scalpel. Then, two enzymatic digestions were performed at 18 °C with 0.125% collagenase type II (Sigma-Aldrich). The fragments obtained were washed, plated in 10 cm plates with growth medium (GM) composed of Dulbecco’s Modified Eagle Medium (DMEM) supplemented with 10% fetal bovine serum (FBS) and 1% antibiotic / antimycotic solution (A/A) and incubated at 23 °C in 2.5% CO_2_. After a week, the bone fragments were removed from the cultures and the adherent cells were collected by treating them with 0.25% trypsin-EDTA (Invitrogen, Alcobendas, Spain). The resulting cell suspension was used to generate several subcultures and cells were maintained for a maximum of 10 passages.

For the experiments, cells were trypsinised, suspended in GM, counted and plated in 6-well plates at a density of 10^5^ cells per well. The next day (day 0), the media was changed and the cells were grown either under control (GM) or mineralizing conditions, using an osteogenic medium (OM; GM supplemented with 50 μg/ml of L-ascorbic acid, 10 mM β-glycerophosphate and 4 mM CaCl_2_) or were induced to differentiate into adipocyte cells using an adipogenic medium (AM, GM supplemented with 10 μg/ml insulin, 0.25 μM dexamethasone, 0.5 mM 1-methyl-3-isobutylxanthine (IBMX) and 5 μl/ml lipid mixture, which contained cholesterol and fatty acids from cod liver oil). Cultures were maintained for up to 20 days and the media was replaced every 3–4 days. Samples for gene expression analysis consisted of 3 replicates each composed of a pool of 2 wells / primary culture that were collected into 1 ml of TRI reagent (Applied Biosystems, Alcobendas, Spain) at days 5, 10, 15 and 20 and stored at -80 °C. Cultures were repeated in 5–8 independent experiments. Development of the cells under GM, OM and AM conditions was monitored using an Axiovert 40C inverted microscope (Zeiss, Germany) and images were captured with a Canon EOS 1000D digital camera.

#### Myocyte gilthead sea bream primary culture

Primary cultures of gilthead sea bream muscle satellite cells were performed as previously described [60]. In brief, the epaxial fast-twitch/white skeletal musculature of juvenile fish (11–23 g) was collected and mechanically disrupted before enzymatic digestion at 18 °C with 0.2% collagenase type Ia (Sigma-Aldrich), followed by 0.1% trypsin (Sigma-Aldrich). Cells were washed in phosphate buffered saline (PBS), resuspended in GM, counted and plated at a density of 1.5–2 × 10^6^ cells per well in 6-well plates and incubated at 23 °C in 2.5% CO_2_.

Samples for gene expression analysis consisted of 3 replicates per sample point and each sample was composed of a pool of 2 wells / primary culture. Samples were collected into 1 ml of TRI reagent (Applied Biosystems) at days 2, 4, 8 and 12 in 4 independent experiments and were stored at -80 °C. For cell culture characterisation, images of the cells at different time-points during the experiment were captured with an Axiovert 40C inverted microscope (Zeiss) coupled to a Canon EOS 1000D digital camera.

#### RNA extraction and cDNA synthesis

Total RNA from gilthead sea bream tissues snap frozen in liquid nitrogen was extracted using a Maxwell^**®**^ 16 MDx Instrument (Promega, Madrid, Spain) with a Maxwell 16 Total RNA Purification Kit (Promega). RNA from cell culture samples was extracted with TRI reagent (Applied Biosystems) according to the manufacturer’s instructions. The quality and integrity of total RNA was verified using a NanoDrop1000 Spectrophotometer (Thermo Scientific, Alcobendas, Spain) and by running it on a 1.5% (m/v) agarose gel before treatment with 1.5 U DNAse (Ambion DNA-*free*™ kit, Austin, Texas, USA). DNA free, total RNA (500–1000 ng) was used for first strand cDNA synthesis in a 20 μl reaction volume containing 100 mM p(dN)6 random hexamers (GE Healthcare, UK), 100 U of RevertAid™ Reverse Transcriptase (Fermentas, Lithuania) and 8 U of RiboLock™ RNase Inhibitor (Fermentas). cDNA was synthesized for 10 min at 20 °C, followed by 50 min at 42 °C and 5 min at 72 °C and the quality was checked by amplifying ribosomal protein S18 (*rps18*) using the following cycle: 10 min at 95 °C, followed by 25 rounds of amplification of 30 s at 95 °C, 30 s at 60 °C and 30 s at 72 °C and finally one cycle of 5 min at 72 °C (*rps18* primer sequences have previously been reported [61]). The PCR products were sequenced and run on a 1% (m/v) agarose gel to confirm amplicon identity and size, respectively.

#### Quantitative real-time PCR (qPCR)

qPCR was carried out in duplicate 10 μl reactions of 1x SsoFast-Evagreen Supermix (BioRad) containing cDNA (≈ 16.7 ng) and 300 nM of forward and reverse primers. Specific PCR primers were designed for gilthead sea bream *ogn* transcripts using Primer premier (Biosoft, Palo Alto California, USA) (Table 1) and those for *ef1α*, *rps18* and *ß-actin* have previously been reported [61]. Quantification was performed in a StepOnePlus thermocycler (Applied Biosystems) using the standard-curve method (software StepOne™ Real-Time PCR Software v2.2) and the following program: 30 s at 95 °C, 45 cycles of 5 s at 95 °C and 15 s at 60 °C. Negative controls were also run and included a no template control (NTC, cDNA was substituted with water in PCR reactions) and a no reverse transcriptase control (RTC, RT was omitted from the cDNA synthesis reaction). A standard curve relating initial template quantity to amplification cycle was generated using serial dilutions of known concentrations of the target template. The templates for the standard curves were generated by conventional PCR using standard conditions, 10 ng cDNA, 1.5 U of Taq polymerase (Readymix Taq PCR Reaction Mix, Sigma-Aldrich) and 200 nM of long-forward and long-reverse primers (Table 1) in a final volume of 50 μl. PCR products were all sequenced to confirm reaction specificity and PCR products for standards were column purified (Illustra™ GFX™ PCR DNA and Gel Band Purification Kit, GE Healthcare) and quantified (NanoDrop1000; Thermo Scientific).

**Table 1:** List of primers used for gene expression analysis by quantitative real-time PCR (qPCR)

Gene name	Primer sequence (5’→3’)	Amplicon (bp)	Ta (°C)	Efficiency	R^2^
*ogn1*	F: GAAGTCTCTCTTATTCACCTGT	138	60	92.4	0.99
	R: CAAAGGGTCACTGAAGTATCCA				
*ogn2*	F: TGTTATTCTCCCATGGATCCTG	125	60	100	0.99
	R: GATCCCCCGCTGCATCTGTGG				
*ogn1*	F: GAAGTCTCTCTTATTCACCTGT	544	60	na	na
	R: GTTGTTGGCATTGAAGGAT				
*ogn2*	F: ATGATGCAACTGAGGACTTTAA	392	60	na	na
	R: GCTCCATCTTCAATCTCAG				
*op*	F: AAAACCCAGGAGATAAACTCAAGACAACCCA	153	68	95.3	0.99
	R: AGAACCGTGGCAAAGAGCAGAACGAA				

Gene name, primer sequence, amplicon length (base pairs, bp), annealing temperature (Ta, ºC) and efficiency (%) and R^2^ are indicated for each primer pair. For *ef1α*, *rps18* and *ß-actin*, the sequences and specific conditions have previously been reported [61]. *F* forward, *R* reverse, *na* not applicable. The longer *ogn* amplicons were used to generate the standards for qPCR

The relative expression of the analysed genes was estimated using the geometric mean of the reference transcripts *ef1α* and *rps18* in the case of cell cultures, since their expression did not vary significantly (*p* > 0.05) between samples. The results of gene expression analysis were expressed as relative expression (copy number) for the cell cultures.

#### Statistical analysis

Significant changes in transcript abundance in the gilthead sea bream tissue panel were tested using a One-way ANOVA with a Bonferroni multiple comparison post-test. A Two-way ANOVA followed by a Fisher’s least significant difference (LSD) post-test was performed using StatPlus:mac LE v5 2015 (AnalystSoft Inc., USA) to identify significant differences in gene transcript abundance in osteoblast and adipocyte derived MSC cell cultures. In myocyte cultures, One-way ANOVA followed by a Tuckey test was performed to detect differences in expression across time. The significance cut-off was set at *p* < 0.05 for all the statistical analysis performed. Data are presented as the mean ± standard error of the mean (sem).

## Results

### Multiple sequence alignments and protein characterization

Analysis of available teleost genomes revealed that they all contain duplicate *ogn* genes and the deduced proteins shared between 65 and 72% amino acid sequence similarity (Additional file 3). In the ray-finned fish lineage lepisosteiformes, the spotted gar contained a single *ogn* gene that encoded a protein that shared 68% amino acid sequence similarity with teleost Ogn1 and 2 suggesting that the teleost specific whole genome duplication generated the 2 teleost *ogn* genes.

**Fig. 1:**
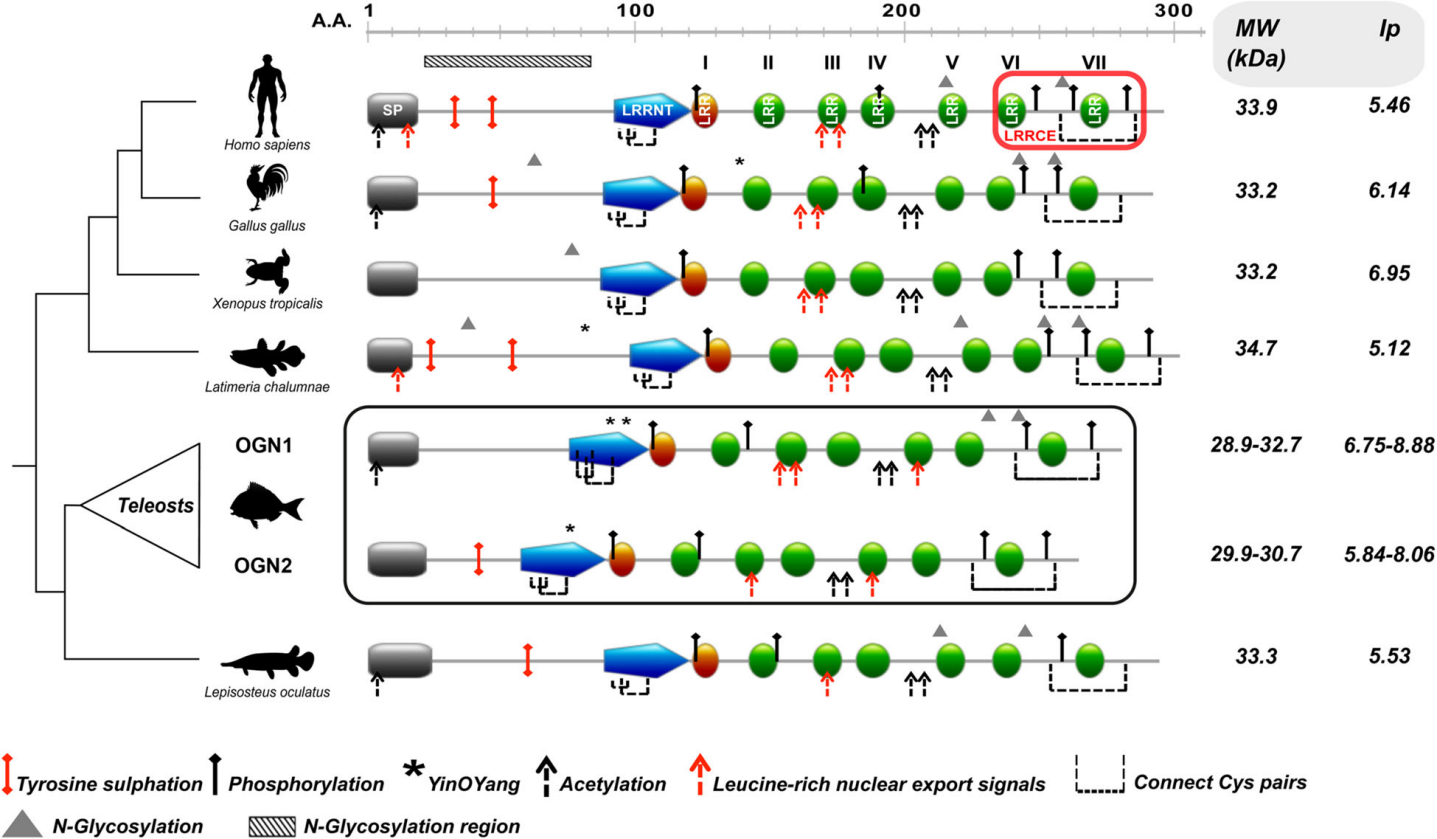
Dendrogram comparing OGN structural features from representative organisms of the main vertebrate lineages. Structural domains/motifs present in the amino acid sequences of OGNs from representative organisms are indicated as coloured blocks using the human OGN sequence as the reference. SP: signal peptide; LRRNT: leucine rich repeat N-terminal motif (blue); LRR: leucine rich repeat motif (green and orange and numbered I-VII); LRRCE: ear-containing leucine-rich repeat C-terminal motif (red box). LRRNT is the N-terminal cysteine flanked capping motif rich in hydrophobic amino acids. Green blocks represent the consensus region for LRR (LXXLXLXXNXL, where L is a hydrophobic amino acid, N is Asn, and X is any amino acid) and the orange block is an incomplete LRR lacking the consensus hydrophobic amino acid. LRRCE is the C-terminal cysteine flanked capping motif. The predicted consensus sites for post-translational modifications (PTMs) are indicated and described in the legend as: tyrosine sulphation (red pin); phosphorylation (black pin); N-linked glycosylation (triangle); *O*-linked glycosylation region (horizontal bar with black blades oblique); Yin O Yang sites (asterisks); acetylation (black dashed arrow). Broken lines connect adjacent cysteine pairs and the leucine-rich nuclear export signal is indicated (red dashed arrow). The consensus sites for disulphide bonds, are present in the N-terminus (C1 - C3 and C2 - C4, *aka* LRRNT capping motif) and C-terminus (C5 - C6, *aka* LRRCE capping motif) of fish Ogn. The scale above the sequences indicates the amino acid (A.A) position. The in silico analysis of the molecular weight (MW, in kilodaltons, kDa) and the isolelectric point (Ip) of analysed OGNs are indicated on the right hand side of the figure. For simplicity the figure only reports the maximum and minimum predicted MW/Ip values. The accession numbers of the sequences used for structural analysis are given in Additional file 1

Transcripts encoding two *ogn* genes were identified in the gilthead sea bream muscle, vertebra and gill arch transcriptomes (Genbank accession numbers: KM603667 and KM603668 for *ogn1* and *ogn2*, respectively). A multiple sequence alignment of gilthead sea bream Ogn1 and 2 with Ogn from other teleosts, non-teleost fish, amphibians, birds and human revealed a conserved signal peptide sequence and seven characteristic LRR motifs typical of class III SLRP family members (Fig. 1 and Additional file 4). The consensus sequence for LRR (LXXLXLXXNXL, where L is a hydrophobic amino acid, N is Asn, and X is any amino acid) was found in LRRs 2, 3, 4, 5, 6, 7 while LRR1 was incomplete and lacked the hydrophobic amino acid at the first consensus site. The central LRR domain was flanked by an N-terminal LRR that incorporated a cysteine-rich cluster (CX_2_CXCX_6_C). The C-terminus contained two cysteine residues (CX_33_C) that flanked an LRR consensus sequence. Additional features shared with other vertebrate class III SLRPs were also identified in fish Ogn1 and 2 proteins (Fig. 1 and Additional file 4).

### Vertebrate SLRP phylogenetic analysis

Phylogenetic analysis of the vertebrate class III SLRPs revealed 6 main clusters, which contained genes from representatives of the fish species used in the analysis (Additional file 5). Specifically, ECMX (extracellular matrix protein X) and ECML (extracellular matrix protein L) were two independent branches of one cluster. No genes for ECMX and ECML were identified in the shark and ECML genes were only found in teleost fish. ECM2 (extracellular matrix protein 2) was duplicated in teleosts and the shark (*C. milli*) and it was the sister group of the ECMX/L clade. A further gene cluster that was more like the ancestral gene, ECM2L, was identified in vertebrates but it only contained genes from fish including the shark.

The other main branches of the phylogenetic tree contained multiple gene clusters, each of which contained genes from each of the representative species used in the phylogenetic analysis. One branch contained DCN (decorin), BGN (biglycan), ASPN (asporin) and NPC (nephrocan), clusters. NPC was absent from teleost fish genomes but present in the shark, spotted gar and coelacanth. Another branch contained FMOD (fibromodulin), LUM (lumican), LUML (lumican-like), KERA (keratocan), PRELP (proline and arginine rich end leucine rich repeat protein) and OMD (osteomodulin) each clustered on a sub branch. A further branch contained PODN/L (podocan and podocan-like) and TSKU (Tsukushi, small leucine rich proteoglycan) sub clusters. A further branch contained CHAD (chondroadherin) and CHADL (chondroadherin-like) clusters (Additional file 5). A characteristic cysteine-like cluster occurred in the N-terminal LRR in the deduced protein of all SLRPs identified. Interestingly, although the cysteine motifs were well conserved for each SLRP member across the vertebrates, the motifs were not conserved between SLRP members belonging to the same class (e.g. in class I and class IV) (Additional file 6).

OGN clustered in the SLRP tree within the branch containing the OPTC (opticin) and EPYC (epiphycan) genes from vertebrates (Additional file 5). The members of the OGN/OPTC/EPYC clade all contained a characteristic cysteine-like cluster (Cx2CxCx6C) in the N-terminal LRR (Additional file 6). The cysteine-like cluster in OGN was also found in ECMX and ECM2 despite their phylogenetic distance. Of the vertebrate SLRP family only FMOD, OGN and ECM2 were duplicated in teleosts.

### OGN phylogenetic analysis

**Fig. 2:**
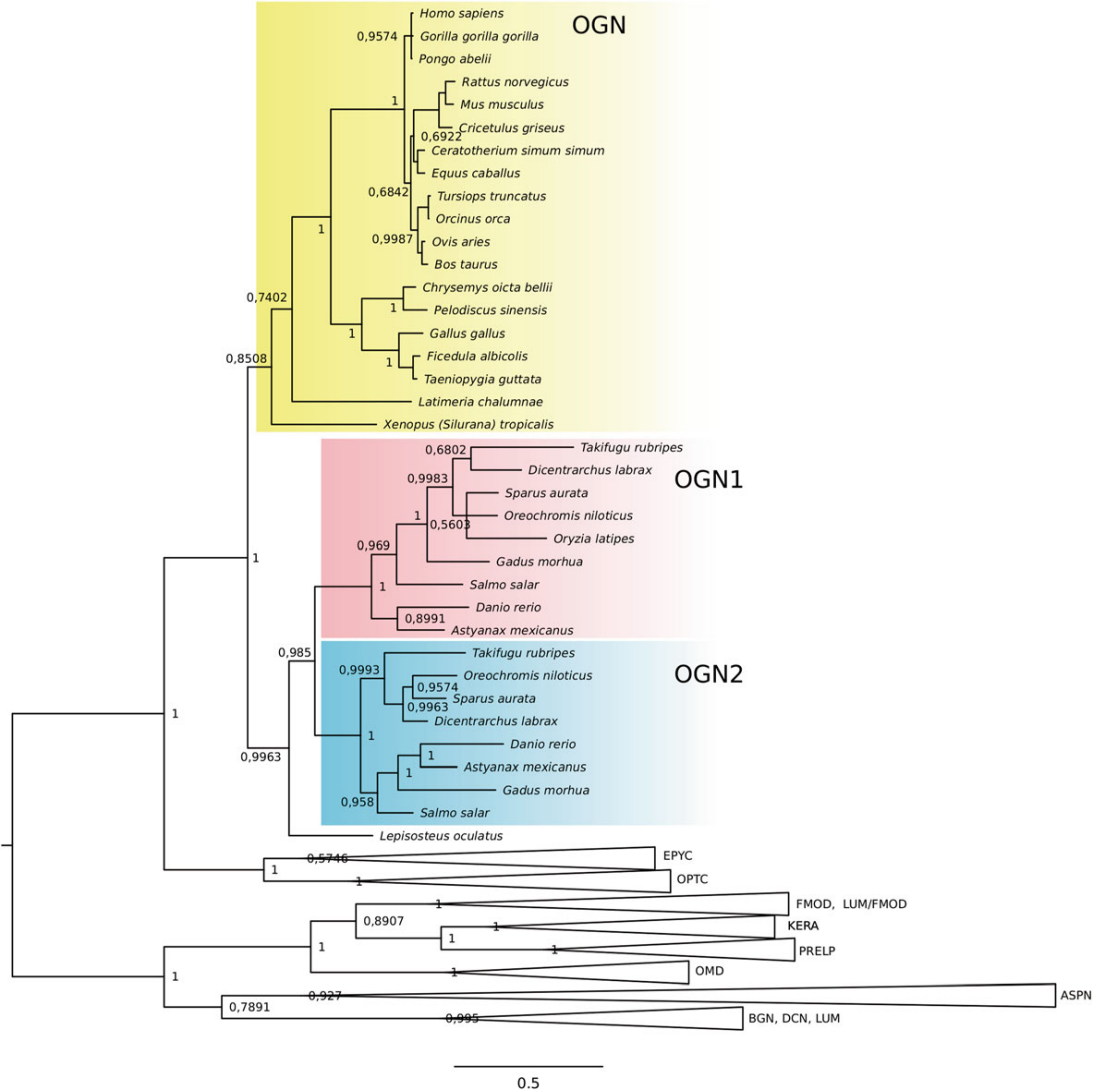
Phylogenetic relationship of osteoglycins (OGNs) in vertebrates. Phylogenetic analysis was performed using Bayesian inference and the tree built in MrBayes 3.2 and branch support values (posterior probability values) are shown for the major protein family clades. All other SLRP family members were used to root the tree. The accession number of all the sequences used in this phylogenetic tree are shown in Additional file 1

Duplicate genes for Ogn only existed in teleost genomes and presumably arose during the teleost specific whole genome duplication (Fig. 2). The BI phylogenetic tree had two major OGN clusters, one cluster contained the ray-finned fish Ogns and the other contained OGN from the terrestrial vertebrates and the coelacanth (Fig. 2). The teleost Ogns clustered into an Ogn1 and Ogn2 clade and confirmed the identity assigned to the gilthead sea bream *ogn1* and 2 cDNAs isolated in this study.

Ogn from the gar (*Lepisosteus oculatus*) was outside the teleost specific Ogn1 and 2 clades. A single OGN homologue was identified in placental mammals, ungulates, rodents, birds, reptiles and amphibians, and in the ancestral fish in the tetrapod lineage, the coelacanth (*Latimeria chalumnae*). The *ogn1* gene from aquatic organisms (teleost fish, coelacanth and turtles) was under positive selection at several amino acid positions (Additional file 7).

### Gene-linkage of *ogn*

**Fig. 3:**
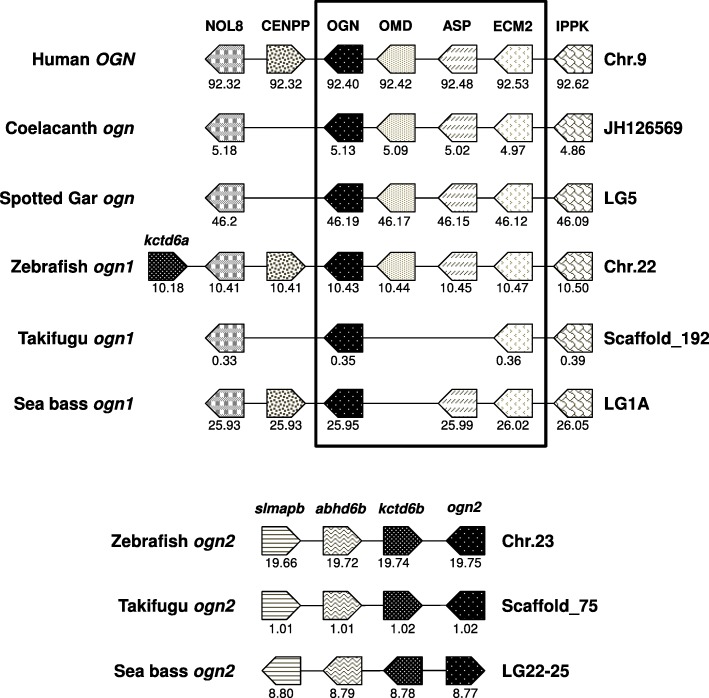
Conserved synteny in vertebrate osteoglycins (*ogn*). The gene environment of *ogn* genes was obtained from the Ensembl Genome Browser and from the UCSC genome browser of the sea bass genome at http://seabass.mpipz.mpg.de. Horizontal lines represent the chromosome fragments and arrow boxes indicate genes and the arrowhead points in the direction of the predicted gene transcription. Homologue genes between species are the same colour (shading) to facilitate perception of conservation. The predicted location of the genes in the chromosome is indicated below each box, in megabase pairs. Note the higher synteny between *OGN* in terrestrial vertebrates and teleost *ogn1*. NOL8 – Nucleolar protein 8; CENPP – Centromere protein P; OGN – Osteoglycin; OMD – Osteomodulin; ASP – Asporin; ECM2 – Extracellular matrix protein 2; IPPK – Inositol 1,3,4,5,6-pentakisphosphate 2-kinase; *kctd6* – Potassium channel tetramerisation domain containing 6; *Slmapb* – Sarcolemma associated protein b; *abhd6b* – Abhydrolase domain containing 6

The gene-linkage of *ogn* revealed highly conserved synteny between fish *ogn1* and tetrapod *OGNs* suggesting that it is most like the ancestral form (Fig. 3). In contrast, the gene-linkage of fish *ogn2* only shared synteny with fish homologues and in zebrafish *ogn1* and *ogn2* had a single common gene in linkage, namely the duplicated potassium channel tetramerisation domain containing 6 genes (*kctd6a* and *kctd6b*).

### Tissue distributions of *ogn1* and *ogn2*

BLAST searches against the EST database in GenBank revealed that transcripts of *ogn1* were present in the olfactory epithelium, eye, muscle, thyroid, skin, bone, scales and digestive tissue while transcripts for *ogn2* were detected in jaw, thyroid, thymus, head kidney, spleen and skeletal muscle of several teleosts (*C. auratus*, *D. rerio*, *G. aculeatus*, *S. salar*, *G. morhua*, *O. niloticus*, *Haplochromis sp.* and *D. labrax*) (Additional file 8).

**Fig. 4:**
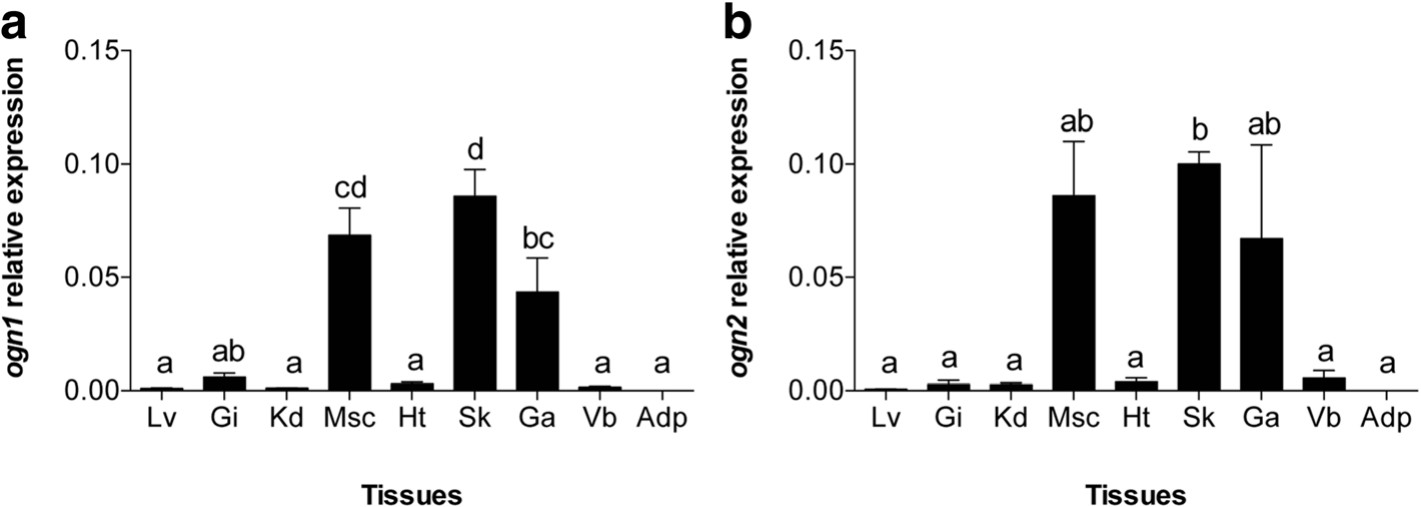
Expression profile of gilthead sea bream *ogn1* and *ogn2* in adult tissues. Quantitative relative expression of (**a**) *ogn1* and (**b**) *ogn2* in adult gilthead sea bream tissues. Lv: Liver; Gi: gill filaments; Kd: kidney; Msc: muscle; Ht: heart; Sk: skin; Ga: gill arches; Vb: vertebra; Adp: adipose. Results are presented as mean ± sem (*N* = 3). Relative expression was determined using the geometric mean of the reference genes *rps18* and *ß-actin*. A One-way ANOVA followed by a Tukey test was used to test for significant differences in transcript abundance between gilthead sea bream tissues that are indicated with letters (different letters denote significant differences, *p* < 0.05). Note that muscle, skin and gill arches have the highest relative expression of both *ogn1* and *2* in gilthead sea bream

Analysis of *ogn1* and *ogn2* transcript distribution in gilthead sea bream tissues using qPCR corroborated the EST analysis and revealed that *ogn1* and *ogn2* were highly expressed in muscle, skin and gill arches but were of low abundance in liver, gill filaments, kidney, heart, vertebra and adipose tissue (Fig. 4). Head kidney, jaw, thyroid, thymus, spleen, olfactory epithelium, eye and digestive tissue were not analysed by qPCR.

### *Ogn* promoter analysis

**Fig. 5:**
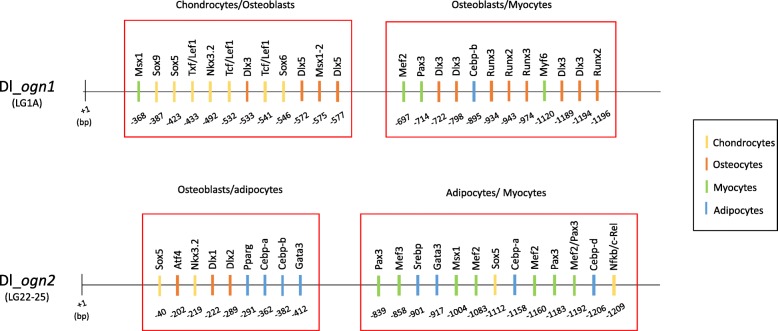
Promoter transcription factors in *ogn*1 and *ogn*2 genes. Approximately 1.2 kilobases of the proximal promoter sequences of the *Dicentrarchus labrax* (Dl) *ogn1* (located in linkage group 1A) and *ogn2* (located in linkage group 22–25) are represented in the figure (solid line). Each vertical rectangle represents a transcription factor binding site predicted with the Matinspector software and below each rectangle the distance of the binding site relative to the beginning of the open reading frame (+ 1 base pairs-bp) is also shown. Red rectangles delimit the regions of the promoters that were enriched in chondrocyte, osteoblast, myocyte and adipocyte specific binding sites. To facilitate identification the different transcription factor binding sites for each cell type are represented in different colours as indicated in the colour chart

UV-responsive transcription factor binding sites have been reported in the human *OGN* promoter. UV-responsive binding sites were identified in the fish *ogn* promoters: three in *ogn1* (Oct1, Isre and p53 at − 327, − 446 and − 473 bp, respectively) and two in *ogn2* that lost the Isre transcription factor binding sites (Oct1, p53, Oct1 at − 939, − 1090 and − 1109 bp, respectively) (Fig. 5).

Comparison of the transcription factor binding sites in the promoters of sea bass *ogn1* and *2* highlighted that the regulation of these genes may explain their different functions in common tissues (Fig. 5). In the *ogn1* 1.2 Kb promoter sequence, we found two blocks (one located at − 368 to − 577 bp and a second at − 697 to − 1196 bp). The former block was enriched in chondrocyte/osteoblast transcription factor binding sites (e.g. Sox5, Sox6, Sox9, Tcf/Lef1, Nkx3.2 and Dlx3, Dlx5, Msx1–2, respectively) and the later in osteoblast/myocyte transcription factor binding sites (Dlx3, Runx2, Runx3 and Mef2, Pax3, Myf6, respectively).

In the *ogn2* promoter region two main regulatory blocks were identified, one located between − 40 to − 412 bp and the second at − 839 to − 1209 bp. The promoter was enriched in osteoblast/adipocyte transcription factor binding sites (e.g. Atf4, Dlx1, Dlx2 and Pparg, Cebpa, Cebpb, Gata3, respectively) and in adipocytes/myocyte transcription factor binding sites (Srebp, Gata3, Cebpa, Cebpd and Mef2, Mef3, Pax3, Msx1, respectively) (Fig. 5).

### Expression of *ogn1* and *ogn2* in bone-derived MSCs primary cultures

**Fig. 6:**
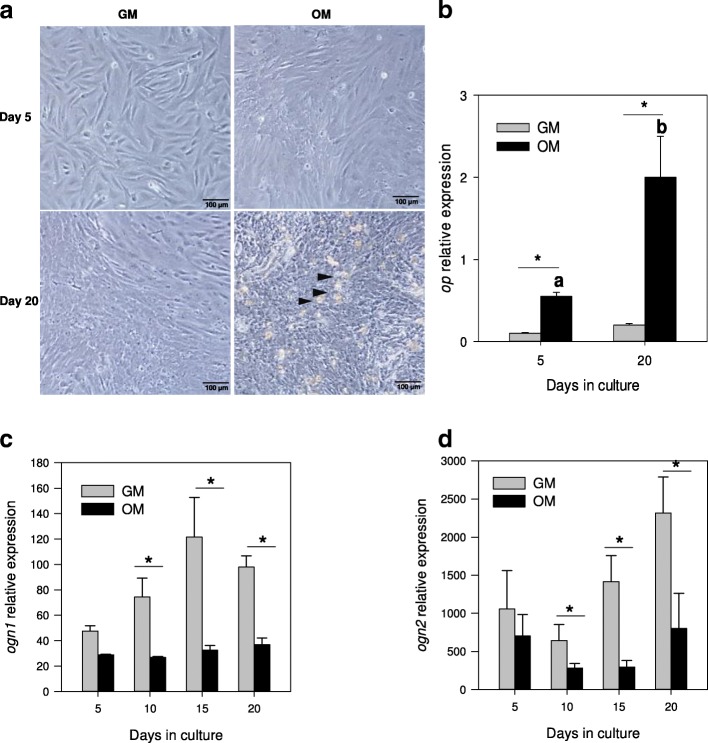
Expression profile of *ogn1* and *ogn2* in gilthead sea bream bone-derived primary MSC cultures in osteogenic conditions. (**a**) Representative images of gilthead sea bream cells derived from vertebra growing in control (GM) or osteogenic (OM) conditions at days 5 and 20 of culture development. The arrowheads indicate nodules of mineralization. The images were acquired using a 20x magnification and a scale bar is also indicated in the image (100 μm). Normalized expression of (**b**) *op*; (**c**) *ogn1* and (**d**) *ogn2* in bone-derived cells growing in GM or OM at different days of the culture (5 to 20). Results are shown as the mean ± sem (*N* = 5–8 independent cultures). Normalized expression (copy number) was determined using the geometric mean of the reference genes *ef1α* and *rps18*. A two-way ANOVA followed by a Fisher’s Least Significant Difference (LSD) post-test was performed to identify differences in gene expression among the experimental groups. Significant differences (*p* < 0.05) between culture conditions are represented with an asterisk and significant differences in each culture condition across time are represented with letters (different letters denote significant differences, *p* < 0.05)

The transcript levels of the duplicated *ogn* genes was analysed in bone-derived MSC primary cultures growing in GM, OM and AM conditions. The morphological analysis of the cells presented in Fig. 6a showed that cells incubated in GM were mostly shaped like fibroblasts, whereas those in OM had a cobblestone-like appearance, and by day 20 multiple nodules of mineralization existed (indicated by the arrowheads).

In bone derived MSCs cells grown in GM, *ogn1* and *ogn2* expression was detected although significant differences in expression were only detected at days 15 and 20 when compared to day 5 for *ogn1* (*p* = 0.01 and *p* = 0.02, respectively). To determine the osteogenic lineage of cells grown in OM, the expression of the ECM molecule osteopontin (*op*) was determined (Fig. 6b). The expression of *op* in cells grown in OM was significantly higher than in cells grown in GM at 5 and 20 days of culture (*p* < 0.001). *op* expression in cells grown in GM was very low and remained stable at 5 and 20 days of culture. Conversely, the expression of *ogn1* and *ogn2* in cells grown in OM was significantly lower from day 10 to 20 than in GM (Fig. 6c-d) (*p*<0.05 in all comparisons) (Fig. 6c).

### Expression of *ogn1* and *ogn2* during bone-derived MSCs differentiation into adipocytes

**Fig. 7:**
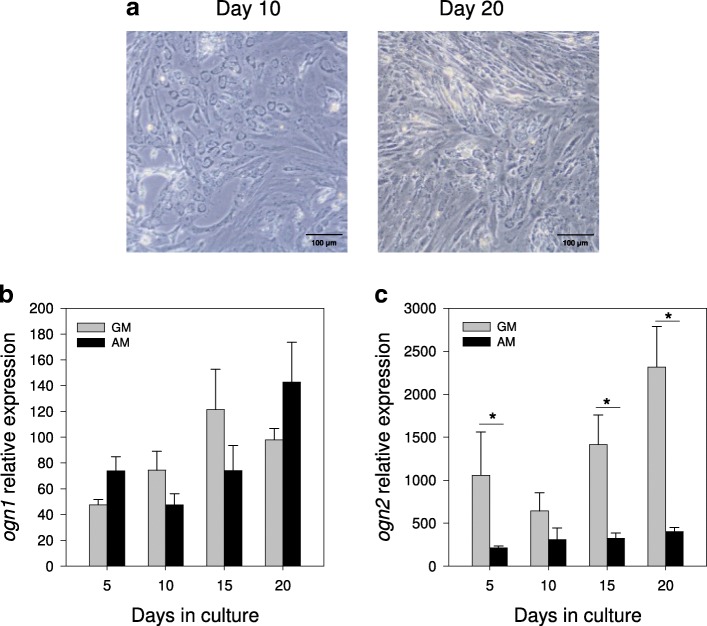
Expression profile of *ogn1* and *ogn2* in gilthead sea bream bone-derived primary MSCs cultures in adipogenic conditions. (**a**) Representative images of gilthead sea bream cells from vertebra growing in adipogenic medium (AM) at days 10 and 20 of culture development. The images were acquired using a 20x magnification and a scale bar is also indicated in the image (100 μm). Normalized expression (copy number) of (**b**) *ogn1* and (**c**) *ogn2* in bone-derived cells growing in control (GM) or AM at different days of the culture (5 to 20) determined using the geometric mean of the reference genes *ef1α* and *rps18*. Results are shown as the mean ± sem (*N* = 5–6 independent cultures). A two-way ANOVA followed by the Fisher’s Least Significant Difference (LSD) post-test was performed to identify differences in gene expression among the experimental groups. Significant differences (*p* < 0.05) between culture conditions are represented with an asterisk

We further studied the role of *ogn* duplicates in the process of differentiation of bone-derived MSCs into adipocyte cells by using specific adipogenic conditions (AM). Morphological analysis of cells cultured in AM (Fig. 7a) revealed that by day 10 they were round and had an enlarged cytoplasm and that by day 20 they contained lipid droplets, which is a characteristic of fully differentiated adipocyte cells. During differentiation, *ogn1* transcript expression was not affected by the medium used (GM or AM) (Fig. 7b) In contrast, the expression of *ogn2* was significantly lower in AM relative to GM (*p*<0.05 in all comparisons) throughout the 20 days of the culture, with the exception of day 10 where no differences were detected (Fig. 7c).

### Expression of *ogn1* and *ogn2* in myocyte primary cell cultures

**Fig. 8:**
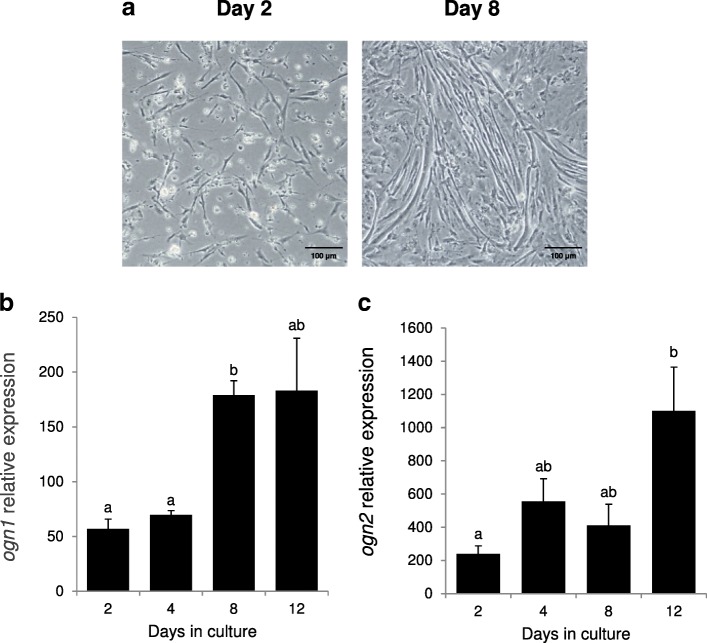
Expression profile of *ogn1* and *ogn2* in gilthead sea bream myocyte primary cultured cells. (**a**) Representative images of gilthead sea bream muscle cells at days 2 and 8 of culture development. The images were acquired using a 20x magnification and a scale bar is also indicated in the image (100 μm). Normalized expression (copy number) of (**b**) *ogn1* and (**c**) *ogn2* in the myocyte cells at different culture days (2 to 12) determined using the geometric mean of the reference genes *ef1α* and *rps18*. Results are shown as the mean ± sem (*N* = 4 independent cultures). A One-way ANOVA followed by a Tukey test was used to test for significant differences in gene expression through time and are indicated with letters (different letters denote significant differences, *p* < 0.05)

Representative images of early myoblasts (day 2) and small myotubes (day 8) are shown in Fig. 8a. *ogn1* expression increased as the cultures progressed and was significantly (*p* < 0.001) higher at day 8 relative to days 2 and 4 and the increase coincided with the time that myocyte cells start to fuse and form small myotubes (Fig. 8a and b). *ogn2* was significantly more expressed at day 12 of the culture relative to day 2 (*p* = 0.023) (Fig. 8c).

## Discussion

OGN is a member of the type III SLRP gene family and clusters with OPTC and EPYC and all members of the cluster had a characteristic cysteine-like cluster, Cx2CxCx6C, in the N-terminal LRR. The sister group of the OGN/OPTC/EPYC clade contained OMD, PRELP, KERA, LUM-L, LUM and FMOD, which were characterised by a Cx3CxCx9C cluster in the N-terminal LRR. *OGN* genes are present in vertebrates, from sharks to mammals and the genome wide gene duplication in teleosts, gave rise to two forms, *ogn1* and *2*. The teleost *ogn1* and *ogn2* gene promoter regions contained common transcription factor binding sites for osteoblasts and myocytes but, while *ogn1* had binding sites that determine expression in chondrocytes, *ogn2* has binding sites that determine expression in adipocytes. Results of in vitro cultures corroborated the promoter analysis and *ogn2* was highly regulated in bone-derived MSC differentiation into adipocytes. The in vitro cell differentiation model and tissue distribution of *ogn1* and *ogn2* taken together with the gene promoter analysis and divergent motifs in the proteins indicate that subfunctionalization of these duplicated proteoglycans probably occurred in teleosts.

### SLRP family evolution

In a recent review and classification of SLRPs published in 2015 [3], eighteen SLRP members were identified in the human genome and were clustered into five different classes: I-V. However, a further three types of SLRP exist, the chondroadherin-like gene (CHAD-L), nyctalopin (NYX) [62] and nephrocan (NPC) [63] and increase the type III family members to twenty-one.

In fish, class I-III SLRP members were identified in their genomes: Class I (ASP, BGN and DCN), Class II (FMOD1–2, LUM1–2, KERA, PRELP, OMD), Class III (OPTC, EPYC, OGN1–2) and three ECM2-like members (ECM2, ECMX, ECMZ) [64]. The present manuscript further expands the list of SLRP in fish to an additional seven members that belong to class IV (NYX, CHAD, CHADL) and Class V (NPC, TSKU and PODN). Analysis of the SLRP members identified in shark, teleosts, spotted gar, coelacanth and mammals provides further insight into the evolution of proteoglycans in vertebrates. All the SLRP members identified in the human genome also existed in the genome of sharks, except for BGN, OPTC and ECMX. Conversely, duplicate LUM genes (LUM1–2) and a third ECM2-like gene (ECMZ) were found from shark to coelacanth suggesting that this proteoglycan was lost in humans. In teleost fish, specific duplications of FMOD, OGN and ECM2 further enlarges the list of SLRP members to twenty-five. Interestingly, we did not find the NPC gene in the genome of teleost fish, although it was present in other fish genomes including the shark, spotted gar and coelacanth. In the human genome the NPC gene is present on chromosome 6 but is an untranscribed pseudogene, but in other mammals (e.g. mice), it is a marker of early kidney and gut development [65, 66]. The loss of NPC in both teleosts and humans may be linked to functional redundancy between members of the SLRP superfamily.

### OGN evolution

Specific gene duplication of *ogn* was confirmed in all the teleost species analysed. High conservation of the gene environment of *ogn1* in teleosts and vertebrates plus the position of teleost *ogn1* in the phylogenetic tree suggests it is the orthologue of human *OGN*. The persistence of duplicate genes in metazoan genomes is quite common [67, 68] and it is assumed to be either a consequence of gain of novel function (neo-functionalization) or partitioning of the function of the ancestral molecule (subfunctionalization). Analysis of the phylogenetic tree coupled with the branch-specific test for positive selection indicated that *ogn1* is evolving under positive selection in the teleosts, which is coherent with a neo-functionalization model for preservation of gene duplicates [69]. Overall, evolution has favoured the conservation of this proteoglycan family, despite the major structural (e.g. presence or absence of ossification) and physiological differences of aquatic and terrestrial vertebrates. Thus, it is conceivable that OGN and other SLRP members play crucial functions that are conserved across taxa and that the gene duplicates in teleosts have acquired new functions. In this context, the presence of conserved and/or novel features in the protein structure and promoter sequences of *ogn* duplicates provides clues about gene function.

### OGN structure

Teleost Ogn duplicates possess most of the key structural motifs that characterize the proteoglycans: a central domain with a variable number of LRRs and a C-terminal domain of poorly defined function. The modular structure and 7 LRRs of class III proteoglycans [70] were conserved in teleost Ogn1 and Ogn2. Analysis of the LRRs in teleost Ogn1 and Ogn2 revealed that the two main repeat units, S (1, 4 and 6) and T (2, 3, 5 and 7) were organised into 4 super-repeats (ST_TSTST) in common with mammalian class III proteoglycans [70]. Conserved clusters of cysteine residues in the N- and C-termini flanked the LRR domains in teleost Ogn1 and Ogn2 and a typical C-terminal leucine-rich repeat cysteine capping motif (LRRCE) was also present and presumably forms 2 disulphide bridges as observed in mammalian decorin and biglycan [64, 71, 72]. The conservation of the LRRs in teleost Ogns suggest it probably has the curved, solenoid structure revealed by the crystal structure of bovine decorin [72]. The general conservation of teleost Ogns with mammalian type III proteoglycans suggests that their basic functions are probably conserved. Although the loss of the N-terminal tyrosine sulphate motif in Ogn1 means post-translational addition of keratan sulphate [15] and the functions resulting from this are unlikely to occur (e.g. in mammals this is the form in the cornea and sclera [73]).

### *Ogn1* and *ogn2* expression in osteoblasts

To assess if teleost Ogn duplicates underwent subfunctionalization, as suggested by the promoter analysis in the present study, we searched for reports of Ogn function in fish. Interestingly, the first indication of sub-functionalization of *ogn* duplicates comes from the gilthead sea bream [26, 27]. In this species, an increase in Ogn protein and *ogn* mRNA levels were associated with the regeneration of scales. However, in these damage - repair models, a combination of hard (scales) and soft tissue damage was also associated with an inflammatory response [26]. Thus, within the first 2 days of regeneration, when the innate immune system responded to close the wound and trigger skin regeneration *ogn1* transcription (but not *ogn2*) significantly increased [26]. As scales only start to form 3–4 days after the injury, this study suggests that *ogn1* (but not *ogn2*) may be a candidate innate immune factor as described for OGN in mammals [17, 18, 74].

Interestingly, we found 2 promoter modules, containing multiple binding sites for transcription factors that are essential for triggering osteoblast differentiation [75–78] in the *ogn1* promoter (but not in *ogn2*), suggesting the subfunctionalization of these duplicates and a prominent role of *ogn1* in this process. In line with this hypothesis, *ogn1* mRNA levels increased significantly, as pre-osteoblasts differentiated into osteoblasts in vitro, but *ogn2* did not change. Interestingly, when terminal maturation of osteoblasts and active bone mineralization occurred *ogn1* and *ogn2* mRNA levels were significantly decreased, suggesting that although these ECM related genes might be involved in the development of the scaffold layers of the bone matrix, they do not appear to be essential for its subsequent mineralization.

### *Ogn1* and *ogn2* expression in myoblast cell cultures

The muscle is a major source of peptides and signalling molecules (a.k.a. myokines) that directly or indirectly regulate bone and cartilage development (for review see [79]). This is the case of OGN that is produced in the skeletal muscle at high levels and have strong bone anabolic effects [80]. In addition, a role for OGN in muscle differentiation has also been shown using mouse myoblast C2C12 cells undergoing skeletal myogenesis [81].

In gilthead sea bream, skeletal muscle *ogn1* and *ogn2* transcripts were highly expressed, although their expression pattern during myocyte differentiation differed. In early stages of myocyte differentiation, the expression of the *ogn* duplicates does not change. However, *ogn1* is up-regulated during myocyte fusion (day 8) and formation of myotubes and *ogn2* is only modified later (day 12), suggesting it may be important in terminal maturation. Interestingly, these differences may be explained by the different organization in *ogn1* and *ogn2* promoters. While the teleost *ogn1* promoter contains a myogenic regulatory factor 4 (Mrf4/Myf6) binding, a key gene involved in triggering muscle differentiation (reviewed in [82]), the *ogn2* promoter contains Mef2 binding sites that enhance expression of specific genes (reviewed in [83]) in the terminal differentiation of muscle cells (reviewed in [84]). In addition, the involvement of Mrf4 [85] and OGN [86] in mammalian muscle cell regeneration has also been described.

Thus, as found in osteoblast cell cultures, *ogn1* (but not *ogn2*) appears to promote or reinforce the phenotype of myocyte cells at early differentiation. The overlapping response of *ogn1* transcription in both cell cultures is particularly interesting because the promoter modules with muscle related transcription factor binding sites are clustered together with osteoblast related modules, namely two key lineage activation markers for driving osteoblast (Runx2) or myoblast (Mrf4/Myf6) cell differentiation from common precursor cells. These coexisting binding sites could reflect the lability of precursor cells that can differentiate into either osteoblasts or myoblasts.

### *Ogn1* and *ogn2* expression in bone derived MSCs differentiation into adipocyte cells

Interestingly, analysis of teleost *ogn* promoters also revealed that in *ogn2*, the myocyte related transcription factor binding sites coexist with a module of adipocyte related binding sites, which are absent from *ogn1*. These transcription factors are responsible for the induction of the central transcriptional regulators of adipocyte differentiation [87] and, upon adipocyte differentiation, for the stimulation of transcription of genes involved in lipid biosynthesis and lipid droplet accumulation within the cells [88]. In this context, we predicted a specific role for *ogn2* in adipose tissue. Expression analysis of *ogn* duplicates in the gilthead seabream bone-derived MSCs undergoing differentiation into adipocyte cells corroborates our predictions as *ogn2* expression was significantly down-regulated during this process. The response of *ogn2* in these cells agrees with those of mammalian cell lines in which adipocyte cell differentiation from bovine bone marrow cells [89], mammalian 3T3L1 pre-adipocyte cell line [90], mouse mesenchymal stem cells (MMSCs) or senile mouse model-derived bone marrow mesenchymal stem cells (SMMSCs) [91] was accompanied by down-regulation of *ogn* mRNA levels. Thus, in the gilthead seabream, ogn2 is the duplicate that appears to have retained the homologous functions in adipose tissue as described for *OGN* in mammalians.

## Concluding remarks

Characterization of the SLRP members from sharks to mammals indicates that the gene family has been conserved since the separation of chondrichthyes and osteichthyes. Few gene duplications of SLRP members occurred even in the teleosts that suffered a specific whole genome duplication. Analysis of the protein structure of *ogn* duplicates and the composition of putative transcription binding sites in their gene promoters support the subfunctionalization of these duplicates, which may have favoured the maintenance of duplicate *ogn* genes in teleosts. Analysis of the *ogn* promoters together with the in vitro cell culture results indicate that *ogn1* is regulated during osteoblast and muscle differentiation (up-regulated during osteoblast differentiation and when myocytes start to fuse and form nucleated myotubes, respectively). Conversely, *ogn2* appears to be an osteoblast lineage specification factor that is severely down-regulated as cells differentiate into adipocytes and also appears to be involved in the later maturation stages of muscle differentiation (when large polynucleated myotubes are formed). Overall our study supports the view that the gene duplicates of *ogn* in teleosts partitioned functions in the case of myocyte differentiation but also acquired specific functions; *ogn1* in osteoblast differentiation and *ogn2* as a candidate inhibitor of adipocyte differentiation.

## Additional files

Additional file 1: Accession numbers of all protein sequences used in this study. (XLSX 16 kb)

Additional file 2: Accession numbers of all nucleotide sequences used in this study. (PDF 14 kb)

Additional file 3: Comparison of vertebrate OGNs and fish duplicates OGN1 and 2. The sequence identity and similarity (between brackets) are shown as percentages and the highest identities and similarities to the gilthead sea bream OGN1 and 2 are shown in bold. Abbreviations: Sa – *Sparus aurata*, Dl – *Dicentrarchus labrax*, Dr. – *Danio rerio*, Lo – *Lepisosteus oculatus*, Lc – *Latimeria chalumnae*, Xt – *Xenopus (silurana) tropicalis*, Gg – *Gallus gallus* and Hs – *Homo sapiens*. (PDF 27 kb)

Additional file 4: Amino acid sequence alignments of human and fish osteoglycins (OGN). The conserved amino acid residues are shaded. The numbers on the right-hand side indicate the position of the amino acid residues. The predicted signal sequences are boxed. The * mark the N-terminal (CX_2_CXCX_6_C) and C-terminal (CX_33_C) cysteine-rich clusters characteristic of OGN proteins and family members. The seven LRR motifs characteristic of class III SLRP family members are marked as L-motif 1–7. The putative glycosaminoglycan-attachment sites (φ) are also marked. The accession numbers of all the sequences used in this alignment are shown in Additional file 1. (PDF 69 kb)

Additional file 5: Phylogenetic relationship of SLRP members in vertebrates. Phylogenetic analysis was performed using the Maximum likelihood (ML) method and the branch support values (posterior probability values) are shown for the major protein family clades. *R. typus* Leucine Rich Repeat and Ig Domain Containing 3 (Lingo3) was used to root the tree. The accession number of all the sequences used in this phylogenetic tree are shown in Additional file 1. (TIF 600 kb)

Additional file 6: Table containing all SLRP members identified in this study. In the table, the general classification (types I-V), the type of N-terminal cysteine-rich cluster present in each SLRP member and the maximum likelihood phylogenetic clusters are also shown. (PDF 27 kb)

Additional file 7: OGNs and positive selection. Summary of tree branches exhibiting signatures of positive selection in gene-trees constructed for vertebrate osteoglycin genes. (PDF 30 kb)

Additional file 8: Digital tissue distribution for *ogn* in teleost fish. Sequence similarity searches (BLASTX and TBLASTN) were done using the gilthead sea bream *ogn1* and *2* sequences against the Expressed Sequence Tags (ESTs) database. (PDF 25 kb)

## Abbreviations

AM: Adipogenic medium
ASPN: Asporin
Atf4: Activating transcription factor 4
BGN: Biglycan
Cebpa: CCAAT enhancer binding protein alpha
Cebpb: CCAAT enhancer binding protein beta
Cebpd: CCAAT enhancer binding protein delta
CHAD: Chondroadherin
CHADL: Chondroadherin-like
DCN: Decorin
Dlx1: Distal-less homeobox 1
Dlx5: Distal-less homeobox 5
DlxX3: Distal-less homeobox 3
ECM: Extracellular matrix
ECM2: Extracellular matrix protein 2
ECM2L: Extracellular matrix protein 2-like
ECMX: Extracellular matrix protein X
ECMXL: Extracellular matrix protein X-like
EPYC: Epiphycan
FMOD: Fibromodulin
Gata3: Gata binding protein 3
GM: Growth medium
Isre: Interferon-stimulated response element
KERA: Keratocan
LRR: Leucine rich repeats
LUM: Lumican
LUML: Lumican-like
Mef2: Myocyte enhancer factor 2
Mef3: Myocyte enhancer factor 3
MSCs: Mesenchymal stem cells
Msx1–2: MSH homeobox 1–2
Myf5: Myogenic factor 5
Myf6: Myogenic factor 6
Myod: Myoblast determining factor
Nkx3.2: NK3 homeobox 2
NPC: Nephrocan
NYX: Nyctalopin
Oct1: Octamer-binding factor 1
OGN: Osteoglycin
OM: Osteogenic medium
OMD: Osteomodulin
Op: Osteopontin
OPTC: Opticin
p53: Tumor suppressor protein p53
Pax3: Paired box 3
PODN/L: Podocan and podocan-like
Pparg: Peroxisome proliferator activated receptor gamma
PRELP: Proline and arginine rich end leucine rich repeat protein
Runx2: Runt related transcription factor 2
Runx3: Runt related transcription factor 3
SLRP: Small leucine-rich proteoglycans
Sox5: Sex determining region Y-box 5
Sox6: Sex determining region Y-box 6
Sox9: Sex determining region Y-box 9
Srebp: Sterol regulatory element binding protein
Tcf/Lef1: Transcription factor/lymphoid enhancer binding factor 1
TSKU: Tsukushi, small leucine rich proteoglycan
