# Supplementary material for: Vertebrate SLRP family evolution and the subfunctionalization of osteoglycin gene duplicates in teleost fish
Source: BMC Evol Biol. 2018 Dec 13;18:191. doi: 10.1186/s12862-018-1310-2 (PMC6293640; doi:10.1186/s12862-018-1310-2)
Supplement: Supplementary file 2 — Accession numbers of all nucleotide sequences used in this study. (PDF 14 kb) [file 12862_2018_1310_MOESM2_ESM.pdf]

Species	Osteoglycin 1 (OGN1)	Osteoglycin 2 (OGN2)
Homo sapiens	KJ891722	-
Gorilla gorilla gorilla	XM_004048267	-
Pongo abelii	NM_001132012	-
Bos taurus	BC102526	-
Equus caballus	XM_001917975	-
Ovis aries	XM_004004076	-
Ceratotherium simum simum	XM_004442726	-
Tursiops truncatus	XM_004313593	-
Orcinus orca	XM_004284119	-
Mus musculus	NM_008760	-
Cricetulus griseus	XM_003505338	-
Rattus norvegicus	XM_006253696	-
Gallus gallus	NM_204209	-
Taeniopygia guttata	XM_002193078	-
Ficedula albicollis	XM_005052906	-
Xenopus (Silurana) tropicalis	ENSXETT00000045198	-
Pelodiscus sinensis	XM_006127563	-
Chrysemys picta bellii	XM_008165394	-
Latimeria Chalumnae	XM_005987054	-
Lepisosteus oculatus	XM_006631102	-
Gadus morhua	ENSGMOT00000003141	ENSGMOT00000004743
Astyanax mexicanus	ENSAMXT00000005237	ENSAMXT00000013872
Danio rerio	NM_001013570	ENSDARG00000031489
Salmo salar	BT056541	BT048437
Oryzias latipes	XM_004086123	-
Dicentrarchus labrax	DLAgn_00097140	DLAgn_00129310
Oreochromis niloticus	ENSONIT00000000620	XM_003438862
Takifugu rubripes	XM_003973017	XM_003963383
