# Supplementary material for: Vertebrate SLRP family evolution and the subfunctionalization of osteoglycin gene duplicates in teleost fish
Source: BMC Evol Biol. 2018 Dec 13;18:191. doi: 10.1186/s12862-018-1310-2 (PMC6293640; doi:10.1186/s12862-018-1310-2)
Supplement: Supplementary file 4 — Amino acid sequence alignments of human and fish osteoglycins (OGN). The conserved amino acid residues are shaded. The numbers on the right-hand side indicate the position of the amino acid residues. The predicted signal sequences are boxed. The * mark the N-terminal (CX2CXCX6C) and C-terminal (CX33C) cysteine-rich clusters characteristic of OGN proteins and family members. The seven LRR motifs characteristic of class III SLRP family members are marked as L-motif 1–7. The putative glycosaminoglycan-attachment sites (φ) are also marked. The accession numbers of all the sequences used in this alignment are shown in Additional file 1. (PDF 69 kb) [file 12862_2018_1310_MOESM4_ESM.pdf]

```
                         Signal sequence
                                                                                                                             *  *  *   ϕ
OGN1_S.aurata      :----MKSLLFTCMLVP-WLAAASARKSDQ-------DSQLIVGSLPGRELNGYFS---DP--LNSRR-----ARRAVS-LADEPDDS--PVSEGG-DASDLPTCLLCVCLIGSVY : 89
OGN1_O.niloticus   :MLDSMKTLFFTCMLVP-WLVAASDRT---------------------KYLDG----------IKPKS-----EKRALP-PADEPDDN--PITAGA-DAADLPTCLLCVCLIGSVY : 74
OGN1_G.morhua      :----MTTVVFLCLLVP-WLATVSGNS------------------------LDQ---------------------KTQLI-LGRVPAGTELDYLES--RKTKLPTCLLCVCLIGSVY : 63
OGN1_D.rerio       :MRTRLRMLIAAVVLVS-MVLSASGAHYHT----------HRHHRLEDVALTEETD---VRAQRNKRSTNGDDDKNALM-LADAPDDSG-PLQPVG-DPSDLPTCLLCVCLIGSVY : 98
OGN2_S.aurata      :-MMQLRTLIFTYVILP-WILSSAAKD---------------------EFMEA----------RKPKPGVVT--YPDY----EEPATD--AAGDP--KADELPTCLLCVCLSGSVY : 72
OGN2_O.niloticus   :-MIPLRILIFTYVMLP-WIVSSAAKG---------------------EYMEA----------RTPAKATVR--ASDYG-ILQDTDTD--GGVPS--KAEVLPTCLLCVCLSGSVY : 75
OGN2_G.morhua      :--MHLEMLICVILIIPSLVLCSPAKN---------------------GYIEA----------RAPKKDIVTIPEFPQD-LPAKPEAAGNMFSTKL-YNSVLPTCLLCVCLIGSVY : 80
OGN2_D.rerio       :-MLDLYATLFTILTHQ-WLLLSCKCS---------------------PVLS-----------KIPKKVILS---QDYD-NPADKDVE--IPGADP-KDDELPTCLMCVCLIGSVY : 74
OGN_L.oculatus     :-MSKLKPLIFSLILVL-WVILAAVTAFTLDS---------EHHNNEKDIFRTPDD---FLTALEAKKQETIIDYEDLPPADTEPAENEVPVLPTPKEAAELPTCLLCVCLIGSVY :101
OGN_L.chalumnae    :---MLHPVLLLFMLVA-WVKAQPVDKDYFTVENVNLDLFDENETGLETTHDVVIYGDYEGKEEPSTRKKESTTPDTFLELLRPPRSPTIEPERVLTQDVDLPTCLLCVCLIGSVY :111
OGN_X.tropicalis   :-MQPLPVCCTLLLLLP-FILSAPSVPQQT-------ISHHEDYIVEGKLFQLNLQ---KMAQTTERNGLTLLSKDRFK-RDQERAGSN-RTAAKV-EDADLPTCLLCVCLSGSVY :100
OGN_G.gallus       :-MKTLQAAFFLVAFVP-LVKFAPPIQQDSPKFYEYVDADFATGSLIQQDYE--ML---PKDTIKDGTNVSL--DTALR-LQADDSEL--SARPTK-DTN-LPTCLLCVCLSGSVY :101
OGN_H.sapiens      :-MKTLQSTLLLLLLVP-LIKPAPPTQQDSRIIYDYGTDNFEES-IFSQDYEDKYL---DGKNIKEKETVIIPNEKSLQ-LQKDEAIT--PLPPKK-ENDEMPTCLLCVCLSGSVY :105

                        L-motif 1              L-motif 2             L-motif 3             L-motif 4          L-motif 5
 *
OGN1_S.aurata      :CEEVSPDMTSVPTLPKETAYLYARFNKIKKISTKDFAGILTLKRIDLTGNLISEIEDGAFSKLALLESLSLAENRLVKLPMLPAKLTSFNANNNFLKTRGVKANAFKKLTKLTHL :204
OGN1_O.niloticus   :CEEVSPDMTSIPTLPKETAYLYARFNKIRKITKTKDFAEIVTLKRIDLTGNLISEIEDGAFSKLTLLEELSLAENQLVKLPALPAKLTSFNANNYNKLKTKGVKANAFKKLTKLANL :189
OGN1_G.morhua      :CEEVSPDMTSVPALPKETAYLYARYNRIKKIRSKDFADILTLKRIDLTGNLISEVEDGAFSKLPVLEELNLSENRLVKLPMLPAKLTVFNANYNLLKTRGVKATAFKKLTRLVNL :178
OGN1_D.rerio       :CEEVDPDMTSVPTLPKETNYLYARYNKIKKITAKDFGDIVTLKRIDFTGNIISEIEDGAFAKLTMLEELSLAENQLAKLPMLPAKLTAFNANHNKLKTKGVKANAFKKLNKLLHL :213
OGN2_S.aurata      :CEEVSPEMSAVPALPKETAYLYARFNKITRIRNSDFADMAPLRRIDLSGNLISEIEDGAFSKLPDLEELILAENKLTRLPIMPTKLVTFNANFNKLKTQGVKATAFKKLTRLSYL :187
OGN2_O.niloticus   :CEDVSPDMSAVPTLPKETAYLYARFNKITKIHREDFADTATLRRIDLSGNLISEIEDGSFSKLPNLEELKLAENRLTKLPMLPSKLVTFNANFNRLKTQGVKANAFKKLTRLAYL :190
OGN2_G.morhua      :CEEVFPQMTAIPALPRETTYLYARFNQINKIKNKDFADMGALKRIDLTGNIISEIEDGAFSKLTNLEELFLAENRLTKLPMLPSKLTTLNANFNLLKSKGVRSNAFKKLPELAFL :195
OGN2_D.rerio       :CEDVSPDMTTVPALPQETAYLYARFNKITKITNKDFANIATLKRIDLSGNLISEIEDGAFSKLSHLEELTLAGNKLVKLPMLPANLLSLDVNHNLLKTKGVKANIFKKLIKLAYL :189
OGN_L.oculatus     :CEEVVPVITAVPALPKETAYLYARYNKIAKIAQKDFSEIPTLRRIDLTGNAISEIEEGAFSKLPQLEELSLAENRLVKLPTLPTKLTLFNANHNRLRTRGVKANAFRKLVNLSYL :216
OGN_L.chalumnae    :CEET--TIDSIPALPQETAYLYARFNKIKSIKTKDFADIPTLKVIDLTGNLIEEIEDGAFSKLLLLEALLAENRLTKLPIALPPKLQLFNANNNRIKNKGIKANAFKKLNNLSYL :224
OGN_X.tropicalis   :CEET--EIEEVPPLPKETAYLYARFNKIKKITAKDFSDFPTLRRIDLTGNLIEEIDDKAFDKLPLLEQLNLAENKITRIPALPTKLTVFNANDNQIKSKGIKANAFKKLTSLAYL :213
OGN_G.gallus       :CEEI--DIEAVPPLPKETAYLYARFNKIKKRIAVSDFADITTLRRIDFSGNMIEEIEDGAFSKLLLLEELSLAENRLVKLPVLPPKLTTFNANQNRIKSRGIKNNAFKKLTNLAYL :214
OGN_H.sapiens      :CEEV--DIDAVPPLPKESAYLYARFNKIKKLTAKDFADIPNLRRLDFTGNLIEDIEDGTFSKLSLLEELSLAENQLLKLPVLPPKLTLFNAKYNKIKSRGIKANAFKKLNNLTFL :218

                     L-motif 6              L-motif 7
                                              *                             *      ϕ
OGN1_S.aurata      :YLADNQLEAVP-QIPDSVQILHLQNNNITEVNVDTFCRSNDT-YLRPSLNEVRLDGNPVVLSKNPDSFTCMKVLPSGRYR- :282
OGN1_O.niloticus   :FLADNMLEAVP-YIPESVRILHLQNNNITEVNVDTFCRSNDTYYLRPSLNEVRLDGNPVVLSKYPDNFTCMKVLPVGKYR- :268
OGN1_G.morhua      :FLADNQLEAVP-QIPESVRTLHLQNNNITEISVDTFCKGNNTYYLRPSLNEVRLDGNPVVLSKYPDSFICLRSLPLGRYY- :257
OGN1_D.rerio       :YLAHNELEAVP-LIPETVRTLHLQNNNISTVSTDTFCKSNDTYYIRPNMNEIRMDGNPINLGQYPNSFICLQSLPIGRYQ- :292
OGN2_S.aurata      :YLGNNELTAVP-QLPESLNVVHLQNNKISTITDETFCKGNTSYYIRTNLYQVRLDGNPIQLSKHPNSFICLETLPIGWYN- :266
OGN2_O.niloticus   :YLGDNELTAVP-HLPESLYVVHLHNNNISTITDETFCKGNTSHYIRTNMYEVRLDGNPLKLSEHPNSFICLQSLPIGWYK- :269
OGN2_G.morhua      :YLGNNKLEVIP-QLPESLQIVHLNNNNINTITDQTFCKGNTTQYIRSTMEEVRLDGNPLVLAQHPNSFICLRNLPTGHYH- :274
OGN2_D.rerio       :YLGDNGLEAIP-PLPESLRVVHLHNNNITSLTDDTFCKGNSTHYIRHNMQEVRLDGNPITLAQHPNSFICLRALPIGHYK- :268
OGN_L.oculatus     :YLANNELEAVPPHLPESLRVLHLQNNNITAITDETFCKGNNTRYIRTNMDEIRLEGNPIILGKYPNSFTCLKSLPMGSYF- :296
OGN_L.chalumnae    :YLTHNVLEKVPPQIPDTLRVLHLQFNNISITDDTFCKANDTSYIRDCMDEIRMEGNPIILAKFPNSFICLKMLPFGRYY- :304
OGN_X.tropicalis   :YLANNQLESVPQNLPESLRILHLQQNGISSITDDTFCKSNDTHYVRSHMDEIRMEGNPVILGKYPNSFICLKTLPSGSYFK :294
OGN_G.gallus       :YLGHNALESVPLNLPESLRILHLQHNNITTINDDTFCKSNNTRYIRTRMDEIRMEGNPILLAKHVNAFSCLRTLPVGTYY- :294
OGN_H.sapiens      :YLDHNALESVPLNLPESLRVIHLQFNNIASITDDTFCKANDTSYIRDRIEEIRLEGNPIVLGKHPNSFICLKRLPIGSYF- :298
```
