# Supplementary figures and images for: Vertebrate SLRP family evolution and the subfunctionalization of osteoglycin gene duplicates in teleost fish
Source: BMC Evol Biol. 2018 Dec 13;18:191. doi: 10.1186/s12862-018-1310-2 (PMC6293640; doi:10.1186/s12862-018-1310-2)

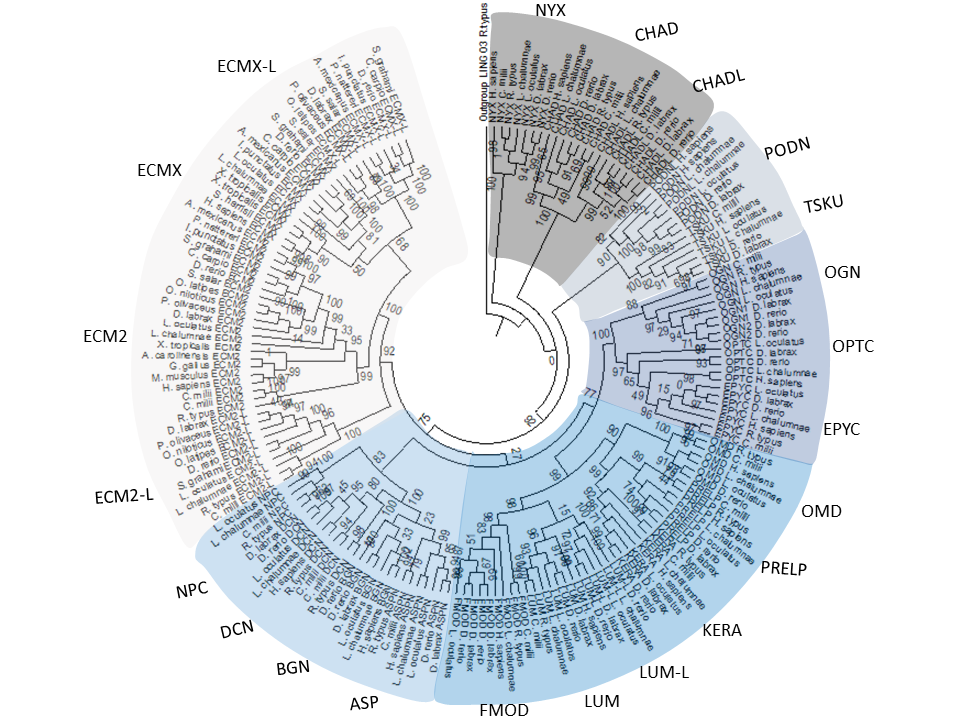

Supplement: Supplementary file 5 — Phylogenetic relationship of SLRP members in vertebrates. Phylogenetic analysis was performed using the Maximum likelihood (ML) method and the branch support values (posterior probability values) are shown for the major protein family clades. R. typus Leucine Rich Repeat and Ig Domain Containing 3 (Lingo3) was used to root the tree. The accession number of all the sequences used in this phylogenetic tree are shown in Additional file 1. (TIF 600 kb) [file 12862_2018_1310_MOESM5_ESM.tif]
