# Supplementary material for: Vertebrate SLRP family evolution and the subfunctionalization of osteoglycin gene duplicates in teleost fish
Source: BMC Evol Biol. 2018 Dec 13;18:191. doi: 10.1186/s12862-018-1310-2 (PMC6293640; doi:10.1186/s12862-018-1310-2)
Supplement: Supplementary file 6 — Table containing all SLRP members identified in this study. In the table, the general classification (types I-V), the type of N-terminal cysteine-rich cluster present in each SLRP member and the maximum likelihood phylogenetic clusters are also shown. (PDF 27 kb) [file 12862_2018_1310_MOESM6_ESM.pdf]

SLRP class	Name	Gene symbol	Cystein motif	ML cluster
I	Biglycan	BGN	CX3CXCX6C	2
I	Decorin	DCN	CX3CXCX6C	2
I	Asporin	ASPN	CX3CXCX6C	2
I	Extracellular matrix protein 2	ECM2	CX2CXCX6C	1
i	Extracellular matrix protein 2-like	ECM2L	CX2CXCX6C	1
I	Extracellular matrix protein X	ECMX	CX3CXCX6C	1
I	Extracellular matrix protein X-like	ECMXL	-	1
II	Fibromodulin	FMOD	CX3CXCX9C	2
II	Lumican	LUM	CX3CXCX9C	3
II	Lumican-like	LUML	CX3CXCX9C	3
II	Prolargin	PRELP	CX3CXCX9C	3
II	Keratocan	KERA	CX3CXCX9C	3
II	Osteoadherin	OMD	CX3CXCX9C	3
III	Osteoglycin	OGN	CX2CXCX6C	4
III	Epiphycan	EPYC	CX2CXCX6C	4
III	Opticin	OPTC	CX2CXCX6C	4
IV	Chondroadherin	CHAD	CX3CXCX8C	6
IV	Chondroadherin-like	CHADL	CX3CXCX8C	6
IV	Nyctalopin	NYX	CX3CXCX5C	6
IV	Tsukushi	TSK	CX3CXCX17C	5
V	Podocan	PODN	CX3CXCX7C	5
-	Nephrocan	NPC	CX3CXCX7C	2
