# Supplementary material for: Vertebrate SLRP family evolution and the subfunctionalization of osteoglycin gene duplicates in teleost fish
Source: BMC Evol Biol. 2018 Dec 13;18:191. doi: 10.1186/s12862-018-1310-2 (PMC6293640; doi:10.1186/s12862-018-1310-2)
Supplement: Supplementary file 7 — OGNs and positive selection. Summary of tree branches exhibiting signatures of positive selection in gene-trees constructed for vertebrate osteoglycin genes. (PDF 30 kb) [file 12862_2018_1310_MOESM7_ESM.pdf]

**Branch**[a]	**Corrected p-value**[b]	**ω^{+c}**	**Pr [$\omega=\omega^{+}$]**[d]
D.labrax ogn1	<0.0001	74.07	0.16
T.rubripes ogn1	0.000	16.40	0.15
O.latipes ogn1	0.001	11.39	0.20
O.niloticus ogn1	0.003	21.47	0.07
Node 15[e]	0.011	863.89	0.04
C. picta belii ogn1	0.020	2785.35	0.02
A.mexicanus ogn1	0.030	654.36	0.09
L.chalumnae ogn1	0.043	12.91	0.19

[a] Branch under episodic diversifying selection at $p \leq 0.05$.
[b] The p-value for episodic selection at this branch corrected for multiple testing using the Holm-Bonferroni method
[c] The ω value inferred for positively selected sites long this branch.
[d] The proportion of sites inferred to be evolving at ω+ along this branch.
[e] Node 15 refers to the separation of *Takifugu rubripes* OGN1 with *Dicentrarchus labrax* and *Sparus aurata* OGN1.
