# Supplementary material for: Vertebrate SLRP family evolution and the subfunctionalization of osteoglycin gene duplicates in teleost fish
Source: BMC Evol Biol. 2018 Dec 13;18:191. doi: 10.1186/s12862-018-1310-2 (PMC6293640; doi:10.1186/s12862-018-1310-2)
Supplement: Supplementary file 8 — Digital tissue distribution for ogn in teleost fish. Sequence similarity searches (BLASTX and TBLASTN) were done using the gilthead sea bream ogn1 and 2 sequences against the Expressed Sequence Tags (ESTs) database. (PDF 25 kb) [file 12862_2018_1310_MOESM8_ESM.pdf]

Species	Tissue	Accession number	Identity	Reference
Carassius auratus	Olfactory epithelium	AM928511	77% *ogn*1	
Danio rerio	Myoblast	CT620959	75% *ogn*1	
Gasterosteus aculeatus	Eyes	DN691791	67% *ogn*1	
Salmo salar	Fast muscle	DY467584	81% *ogn*1	
Salmo salar	Thyroid	EG868446	63% *ogn*1	
Salmo salar	Skin	-	-	Krasnov et al., 2012 doi: 10.1186/1471-2164-13-130
Danio rerio	Fin	EV761307	77% *ogn*1	
Danio rerio	Postcranial axial skeleton	-	-	Kessels et al., 2014, PubMed: 24608635
Danio rerio	Pituitary	-	-	He et al., 2014, doi: 10.1530/JOE-13-0488
Sparus aurata	Skin/scales	-	-	Ibarz et al., 2013, doi:10.1007/s10126-013-9513-4
Gadus morhua	Digestive tissue	FF416230	71% *ogn*1	
Oreochromis niloticus	Skin	GR683533	82% *ogn*1	
Haplochromis sp.	Jaw	BJ695864	75% *ogn*2	
Salmo salar	Thymus	EG772938	72% *ogn*2	
Salmo salar	Thyroid	EG869322	70% *ogn*2	
Salmo salar	Head kidney	EG900788	70% *ogn*2	
Dicentrarchus labrax	Spleen	FM024708	67% *ogn*2	
Oreochromis niloticus	Skeletal muscle	GR688606	83% *ogn*2	
